# Eco-Friendly Fibers Embedded Yarn Structure in High-Performance Fabrics to Improve Moisture Absorption and Drying Properties

**DOI:** 10.3390/polym15030581

**Published:** 2023-01-23

**Authors:** Hyun-Ah Kim

**Affiliations:** Korea Research Institute for Fashion Industry, 45-26, Palgong-ro, Dong-gu, Daegu 41028, Republic of Korea; ktufl@naver.com; Tel.: +82-10-9361-3149

**Keywords:** perspiration absorption, fast-drying, environmentally friendly fiber, high-performance fabric, quadrilobal PET

## Abstract

This study examined the perspiration absorption and drying characteristics of eco-friendly fiber-embedded fabrics with different yarn structures. The wicking and drying rates of fifteen fabrics made from quadrilobal PET, Lyocell, and bamboo fibers were measured using two evaluation methods and compared with the pore diameter and hygroscopic characteristics of the constituent fibers in the yarns. The sheath/core yarn structure played a vital role in improving the moisture absorption and drying properties of the eco-friendly fibers embedded in high-performance fabrics, which was partly affected by the hygroscopicity and non-circular cross-section of constituent fibers in the yarns. Superior perspiration absorption and drying properties among the various eco-friendly high-performance fabrics were observed in the quadrilobal PET/Lyocell sheath/core and quadrilobal PET/bamboo spun yarn fabrics. By contrast, the PET/Lyocell Siro-fil, bamboo spun, and hi-multi PET yarn fabrics exhibited inferior moisture absorption and drying properties. In particular, the evaluated results between transverse and vertical wicking measuring methods in absorption property showed a similar trend. In contrast, the drying property measured between the drying rate (min) at a steady state and the drying rate (g) at a transient state showed a different trend. Multiple regression analysis showed that the wicking property of the eco-friendly fiber-embedded fabrics was mainly related to the pore diameter, cross-sectional shape, and absorption property of the fibers in the yarns, and it was also highly associated with the drying characteristics of the fabrics. The market application of the sheath/core yarn structure using Lyocell and bamboo fibers with quadrilobal PET is available for producing eco-friendly fabrics that can contribute to environmental improvement and wear comfort related to the moisture absorption and fast-drying properties of the woven fabrics.

## 1. Introduction

The environmental impact of energy consumption, pollution, and global warming has attracted considerable attention. The textile industry is imparting some impact on a person as a minor contributor [1]. Recently, eco-friendly fibers have been developed by many fiber companies. The term eco-friendly in the textile area has been coined to define a new fiber and its manufacturing process without harming human beings [2].

Eco-friendly fiber materials are classified into three categories: natural fibers such as organic cotton, Lyocell, and bamboo; biodegradable synthetic fibers, including polylactic acid (PLA); and recycled fibers developed in Japan [2]. Among them, bamboo fibers give excellent wear comfort with good absorption when wearing clothing with 100% biodegradable properties. Lyocell fibers also have good absorption with biodegradable properties. High-performance fabrics for wear comfort allow the human body to provide cooling because of moisture absorption and evaporation by drying behavior. Minimizing perspiration build-up in clothing is critical for comfort while wearing clothing.

Moisture liquid transmission in the fabrics involves a two-stage process: initial wetting and then wicking [3]. The drying process from water absorbed in the fabric is followed after wetting and wicking. Many studies [4,5,6,7,8,9,10] focused on improving wear comfort by sweating have been conducted by combining hydrophobic and hydrophilic yarns. Along with these studies, the absorption and drying properties of fabrics were examined using different measuring methods in terms of yarn and fabric structural parameters, respectively [11,12]. Fan et al. [13,14,15,16,17,18] conducted intensive studies to improve the moisture absorption of different knitted materials using plant-based structures. They developed various knitted fabrics mimicking the branching network of trees to improve water transport properties. On the other hand, the yarn specimens used in their studies were regular yarns made from cotton and polyester. The fabric specimens were limited to knitted structures. In particular, Beskisiz et al. [19] examined the effects of super-absorbent fibers on the drying behavior of knitted fabrics. Eskin et al. [20] introduced new polypropylene (PP) filaments with superior absorption rates to improve the wear comfort of clothing. Prior studies mentioned above reported the improving and enhancing technologies of the water absorption and drying properties of fabrics made from natural and synthetic fibers, such as wool, cotton, PET, and PP. 

Recently, various wear comforts properties of knitted fabrics produced using a new spinning method were reported using eco-friendly fibers, such as modal, hemp, Lyocell, bamboo, and other fibers [21,22]. Of these studies, Kim and Kim [21] examined the wear comfort of knitted fabrics made from PTT (polytrimethylene terephthalate)/Lyocell/cotton mixed air vortex yarns. Kim [22] reported the wicking and drying properties of knitted fabrics made from PTT/wool/modal blended yarns using the ring, compact, and air vortex spinning systems. Zupin et al. [23] identified some parameters with the strongest influence on the water and air permeability of cotton fabrics using the porosity parameters. Some studies [23,24,25,26,27] examined the porosity of functional hollow filaments and their composite yarns and woven fabrics, as well as their effects on water and moisture permeability. 

Several studies have been carried out to improve moisture absorption and drying of fabrics by sheath/core composite yarns [28] or blended yarns composed of hydrophobic and hydrophilic fibers [29]. As specially produced fibers, irregular cross-sectional fiber such as quadrilobal PET was commercialized, and hi-multi microfibers were developed by the splitting spinning method to improve moisture management properties.

Recently, the wear comfort characteristics of the quadrilobal PET/bamboo/Lyocell-included fabrics were investigated, in which the push and pull model for moisture absorption and drying was proposed [30]. In addition, quadrilobal PET/Lyocell core/sheath yarn fabrics suitable for winter warmth clothing have been developed, and bamboo/Lyocell staple yarn fabrics applicable for summer clothing demonstrated improved thermal conductivity and heat retention rate [31]. Kim [1] investigated the moisture vapor permeability of eco-friendly fiber-embedded fabrics made of various yarn structures with several fibers. In prior studies [30,31], however, various evaluation methods related to the moisture and vapor permeable properties were not considered with the deficiency of specimens, even though it is essential to decide on a comfortable feeling while wearing clothing. Moreover, despite these studies, there are few reports on moisture absorption and drying characteristics of woven fabrics made from various yarn structures using quadrilobal PET, Lyocell, and bamboo eco-friendly fibers. In addition, there are no reports on the difference in water absorption and drying properties according to the yarn structure and measuring method. In contrast, the main concern of these studies is how the absorption and drying properties are influenced by yarn structure and constituent fiber characteristics and how these are associated with the pore size of yarns according to the measuring method. Therefore, in this study, seven types of composite yarn specimens are made using Siro, Siro-fil, and sheath/core yarn spinning systems. Eco-friendly fiber materials such as quadrilobal PET, Lyocell, and bamboo are used with PET and PP filaments, which enhance water permeability by passing water and moisture vapor as drainage in the yarns. In addition, two types of wicking rates and drying of 15 fabric specimens made from different yarn specimens are measured and compared in terms of yarn structure and constituent fiber characteristics. Therefore, to overcome these shortcomings in prior studies and based on previous work [1], this study examined the detailed method to improve the perspiration absorption and fast-drying characteristics of the high-performance fabrics according to the evaluation method. Finally, eco-friendly fiber-embedded yarn structures applicable to the market are proposed to improve moisture absorption and fast-drying properties of the high-performance fabric with environmental improvement.

## 2. Experimental Section

### 2.1. Preparation of Yarn Specimens

Seven types of composite yarn were prepared using the ring, Siro, and sheath/core yarn spinning systems. Table 1 lists the specifications of the yarn specimens. Yarn specimens (1), (2), and (3) were used as warp yarns, and yarn specimens (4), (5), (6), and (7) were used as weft yarns. Two existing PET and PP filaments were used for weft yarn insertion. A prior study referred to detailed yarn manufacturing methods [1]. Table 1 lists the twist multiplier (TM), spindle rpm, and blend ratio for the yarn specimens. Yarn mechanical property such as strength and the breaking strain was measured using a Tensorapid III apparatus (Zellweger, Uster, Switzerland) according to KSK 0210.

### 2.2. Fabric Specimens Preparation

Table 2 lists the specifications of fifteen fabric specimens made on a rapier loom (GTX 4-R, Picanol, Belgium). The warp and weft densities of 15 fabric specimens were the same as 36.0 ends/cm and 24.6 picks/cm, respectively. The fabric thickness was measured at a pressure of 2 gf/cm^2^ using a FAST-1 compression meter [1]. The fabric structure was a plain weave pattern. The fabric specimens were classified into three groups (A, B, and C).

Fabric specimens 1 to 5 (group A in Table 2) were prepared using five weft yarn specimens ((4) to (8) * in Table 1) and a fixed warp yarn (PP/Lyocell sheath/core). Group B consisted of five fabric specimens (6 to 10 in Table 2) with five weft yarn specimens ((4) to (8) * in Table 1) and a fixed warp yarn (PET/Lyocell Siro-fil). Group C was comprised of five fabric specimens (11 to 15 in Table 2) with five weft yarn specimens ((4) to (8) * in Table 1) and a fixed Lyocell Siro-spun warp yarn. Yarn specimen (9) * was inserted alternatively in all fabric specimens as a second weft yarn for a plain weave pattern. 

### 2.3. Evaluation of the Pore Size

The main concern of pore diameter measurement is how the constituent yarn structure in the fabric affects pore size, moisture absorption, and drying properties of fabrics. The pore diameter (µm) was assessed using a capillary flow porometer using the ASTM measuring method [32]. The maximum pore diameter (P) was measured and calculated using Equation (1) from the medium value of the graph between airflow and pressure measured using a capillary flow porometer (CFP-1200 AE PMI Co., Ithaca, NY, USA) [1].

P = Cγ/ρ(1)

where P, γ, and ρ are the maximum pore diameter (μm), the surface tension of the liquor (dyne/cm), and pressure (psi), respectively; C = 0.415 when ρ is in psi units. The yarn and fabric cross-sections and surfaces were measured using field emission scanning electron microscopy (FE-SEM S-4100, Hitachi Co., Omori, Japan).

### 2.4. Evaluation of Wicking Rate

Wicking measurement to assess moisture absorption is divided into two methods: transverse and vertical wicking. Transverse wicking assessment is performed using the AATCC 39 measuring method [33] to determine the horizontal moisture permeability. Five fabric specimens, 20 cm × 20 cm in size of each, were prepared. Fifteen to twenty-five water drops per five seconds were dropped on the fabric specimen [30]. A region of the fabric specimen on which the drop falls was illuminated by a light beam to produce a bright reflection from the liquid surface [30]. The elapsed time (second) between the drop reaching the fabric surface and the disappearance of the reflection from the liquid surface was measured and was considered the wicking rate (W_1_, second). A shorter time indicated better wicking of the fabric [34]. 

Vertical wicking was measured using the JIS L 1907 measuring method [35]. This method is called the Bireck method, a measure of vertical perspiration permeability. Five strip specimens, 20 cm × 2.5 cm in size, were prepared for each fabric specimen. The wicking length (W_2_, mm) by the capillary phenomenon after 10 min was measured [31]. The mean value of the warp and weft directions was calculated.

### 2.5. Evaluation of Drying Rate

The drying rate was measured using two methods [36] with moisture regain (M, %) of the fabric. The drying rate (D_1_, min) was measured according to JIS L 1096 [36] using a moisture measuring apparatus (IT-ACD, INTEC Co. Ltd., Tokyo, Japan) [30]. The time (minute) taken until the specimen weight was constant while weighing the fabric specimen in the measuring apparatus was recorded as the drying rate (D_1_, min) [30]. The moisture regains (M, %) of each fabric specimen were calculated using Equation (2).

M (%) = (m_2_ − m_1_)/m_2_ (2)

where M, m_1,_ and m_2_ are the moisture regain (%), initial fabric mass (g), and fabric mass (g) after passing through a mangle, respectively. 

The drying rate (D_2_, g) was assessed using an absorption measuring system (HR 200, Japan). Five specimens, 15 cm × 15 cm in size, were submerged in distilled water for three hours at 27 ± 2 °C in a water bath, and the fabric mass (G_1_) was weighed as an initial fabric mass (g) after removing the fabric from the water bath using an HR 200 system. After ten minutes, the fabric mass (G_2_) was reweighed using an HR 200 apparatus. The evaporated free absorption mass (G_1_–G_2_) was considered as the drying rate (D_2_, g) using Equation (3).

D_2_(g) = G_1_ − G_2_ (3)

where D_2_ is the drying rate (g), G_1_ is the initial fabric mass (g), and G_2_ is the fabric mass (g) after ten minutes. Finally, three drying characteristics were obtained: moisture regain (M, %), drying rate (D_1_, min) as a measure of drying at a steady state, and the drying rate (D_2_, g), i.e., evaporated free absorption mass as a measure of drying at the transient state.

## 3. Results and Discussion

### 3.1. Fabric Pore Diameters According to the Yarn Structure

The fabric porosity has been classified into micro and macro porosities [19,37]. Micro porosity is caused by fine voids among the fibers in the yarns. Micro porosity is associated with the absorption and capillary wicking of fabrics. Macro porosity is caused by the void spaces among the threads. Macro porosity is more important for air permeability, and micro porosity is more applicable to absorption and capillary phenomena. In this study, the theoretical porosity [23,34] could not apply because the fabric specimens used in this study were produced with the same fabric structural parameters, meaning that the theoretical porosity may not be available to examine the difference in the moisture absorption and drying properties among the fabric specimens. Accordingly, the pore size was measured and calculated using the graph measured, which was mentioned previously in the Experimental Section. 

**Figure 1:**
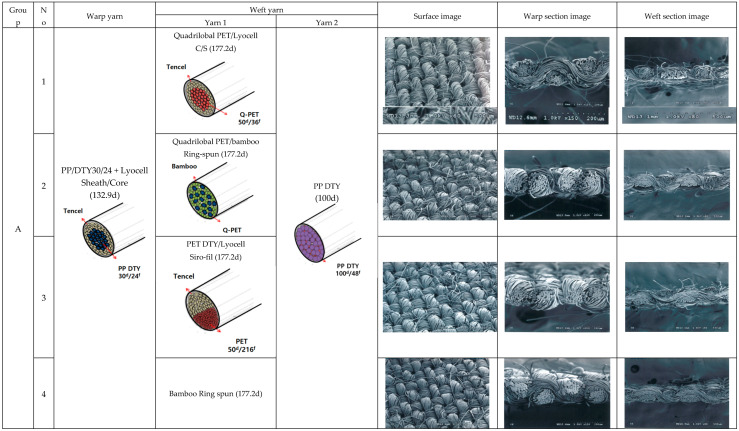

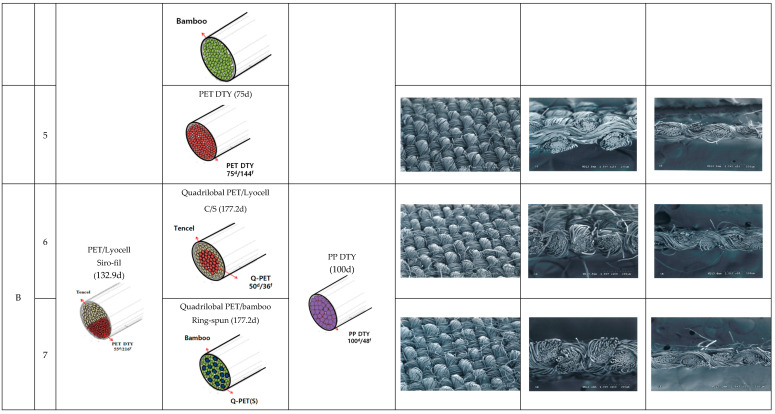

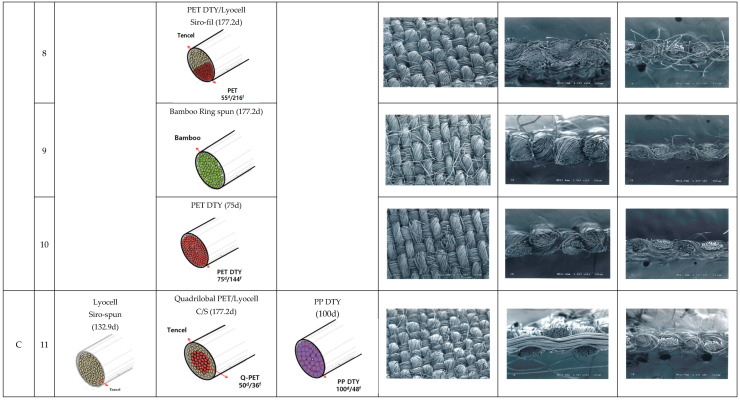

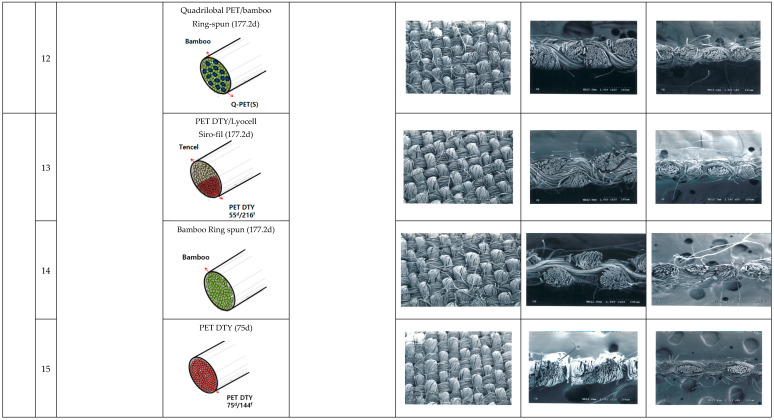
SEM images of surface (×60), warp (×150), and weft(×80) sections of the fabric specimens.

Figure 1 presents cross-sections of the warp and weft yarn specimens and SEM images of the surface and cross-sections (warp and weft) of the fabric specimens, in which schematic diagrams of the yarn specimens were drawn. As shown in SEM images of the fabric surface in Figure 1, the difference in macro porosity among 15 fabric specimens (groups A, B, and C) could not guess because of the same yarn count and fabric set of the 15 fabric specimens. By contrast, the difference in the micro porosity according to yarn structure in each fabric group A (specimens 1 to 5), B (specimens 6 to 10), and C (specimens 11 to 15) was appreciated, as shown in the SEM images of the warp and weft sections of the fabrics in Figure 1. 

In Figure 1, warp section images show SEM images of warp yarns cut perpendicular to the warp direction. The warp section from specimens 1 to 5 (group A) shows PP/Lyocell sheath/core yarns and many air voids along the round-shaped border between core and sheath in the yarn cross-section corresponding to the schematic diagram of the warp yarn. The weft section images show SEM images of weft yarns cut perpendicular to the weft direction, i.e., The SEM image of specimen 1 shows quadrilobal PET/Lyocell sheath/core yarn and PP yarn; quadrilobal PET/bamboo ring-spun yarn and PP yarn for specimen 2; PET/Lyocell Siro-fil yarn and PP yarn for specimen 3; bamboo ring-spun yarn and PP yarn for specimen 4; and PET yarn and PP yarn for specimen 5. These were compared with schematic diagrams of the yarn models. 

Groups B and C show the same fashion as group A with different warp yarns of PET/Lyocell Siro-fil (group B) and Lyocell Siro-spun (group C) with the same weft yarns (five types) as group A. These SEM images were compared with pore diameters measured in the fabric specimens, and absorption and drying properties of fabric specimens were examined in terms of the pore diameter according to various yarn structures. In this study, the measured pore diameter was considered a primary factor affecting the absorption and drying rates of the fabrics, depending on the yarn structure and constituent fiber characteristics. According to a prior study [27], Kim and Kim [27] reported two theoretical porosities, i.e., total porosity (ε) and volume porosity (Pv); of these two porosities, total porosity (ε) was associated with fabric thickness, fabric mass and fiber density, the other theoretical porosity, volume porosity (Pv) was expressed in terms of fabric mass, fabric thickness, yarn diameter, and linear yarn density. In addition, they showed that the theoretical porosity was highly correlated with the measured maximum pore diameter (P). This means that measured pore diameter is mostly dependent on the yarn structure and fabric porosity affecting the absorption and drying properties of the fabrics. 

Table 3 lists the measured wicking and drying rates of the fabric specimens with different measured pore diameters. The deviation in Table 3 denotes the difference between the maximum and minimum values of the experimental data. Statistical analysis (F-test) was conducted to verify the statistical significance of the experimental data (Table 3). F-test to the difference of mean value between each specimen was conducted to verify the significance of the mean values of the four wicking and drying rates, including the pore diameter, with a 95% confidence limit (5% of significance level). The mean value of the specimens of each group for the pore diameter (µm) was statistically significant, as F_0_ (V/Ve) > F(4, 20, 0.95) and *p* < 0.05.

**Figure 2:**
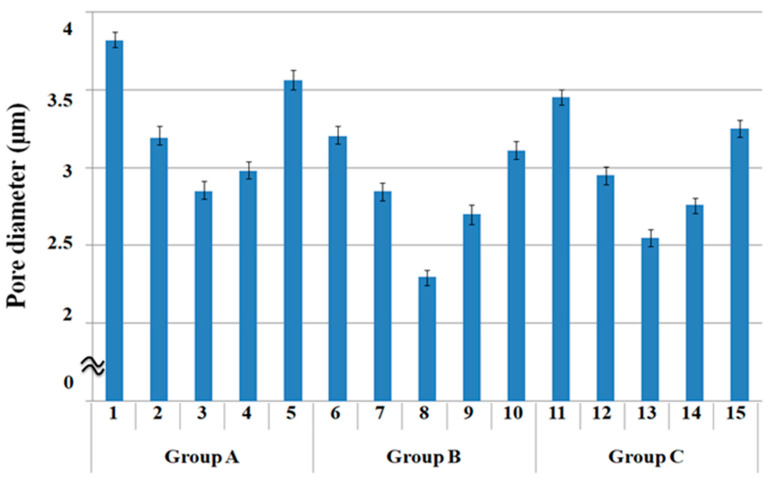
Pore diameters of three fabric groups (*p* < 0.05).

Figure 2 presents a diagram of the pore diameters of the 15 fabric specimens listed in Table 3 [1]. The pore diameters of the three groups of fabrics were compared according to the warp yarn structure. The pore diameters of the five specimens in group A were greater than those in groups B and C because the pore size of the PP/Lyocell sheath/core yarns used as a warp yarn of group A is larger than that of the PET/Lyocell Siro-fil and Lyocell Siro-spun yarns used as the warp yarns of groups B and C. These were verified by SEM and optical microscopy of the constituent warp yarns used in the fabric specimens, which was referred to prior study (Table 6 in [1]).

A prior study [1] referred to optical microscopy and SEM images of the yarn specimens (Table 6 in ref. [1]). Capillary channels of many air voids on the border between the sheath and core in the PP/Lyocell sheath/core yarns (1) were observed (Table 6 in ref. [1]). By contrast, a compact yarn cross-section was observed in the PET/Lyocell Siro-fil yarn (2), and small voids appeared in the Lyocell Siro-spun yarn (3). The capillary channels in the sheath/core yarns (1) allow the moisture absorbed by Lyocell wrapper fibers to move out easily through the PP filaments in the core. PET/Lyocell Siro-fil yarns (2) impede moisture flow in the yarns because of the small pore size and compact yarn structure by the Siro-twist in the yarns (Table 6 in ref. [1]). Lyocell Siro-spun yarns (3) have a similar yarn structure to the Siro-fil yarn by the Siro-twist. In addition, quadrilobal PET/Lyocell sheath/core yarns (4) have same yarn structure as PP/Lyocell sheath/core yarns, i.e., the capillary channels shown in Table 6 (in ref. [1]) between the Lyocell fibers in the sheath and quadrilobal PET filaments in the core enable absorbed moisture and vapor through the sheath to evaporate from the yarns. Many micro-voids in the quadrilobal PET/bamboo ring spun yarns (5) provide drainage channels to remove and evaporate the moisture and vapor absorbed from the human body. 

Regarding the pore size of the fabrics according to the weft yarn structure (Figure 2), of the five types of fabrics in group A, the sheath/core fabric 1 and quadrilobal PET/bamboo spun fabric 2 exhibited larger pore diameters than Siro-fil fabric 3. In addition, the pore diameter of the Siro-fil fabric 3 was smaller than that of the bamboo spun fabric 4 and PET filament fabric 5. These were verified by the SEM images shown in Table 6 [1]. Round-shaped capillary channels in quadrilobal PET/Lyocell sheath/core yarn (4) and relatively large air voids in the quadrilobal PET/bamboo spun yarn (5) were observed, resulting in relatively large pore diameters for fabric specimens 1 and 2 (Figure 2).

By contrast, the compact yarn cross-section was observed in yarn (6) in Table 6 [1], resulting in a small pore diameter for fabric 3 (Figure 2). On the other hand, yarn (7) showed a relatively loose yarn cross-section, similar to yarn (5). The pores in yarn (7) were smaller than those in yarn (5), resulting in fabric specimen 4 having a smaller pore diameter than fabric specimen 2 (Figure 2). Non-twisted parallel filament bundles in the yarn specimen (8) were also observed in the SEM image of the yarn surface, which produces fine capillary channels along the filament bundles with many pores in the yarn cross-section. These were also shown in the SEM and optical microscopy images of the cross-sections of yarn specimen (8) (Table 6 [1]), resulting in a large pore diameter of fabric specimen 5, compared to the Siro-fil and spun yarn fabric specimens 2, 3, and 4. A similar trend to the fabric specimens in group A was observed in fabric specimens in groups B and C. 

On the other hand, according to previous studies [19,37], the absorption and capillary wicking of the fabrics were incorporated with micro-porosity, which is caused by fine voids among the fibers in the yarns. In contrast, macro-porosity is produced by the spaces among the yarns in the fabric. Accordingly, the pore diameters assessed in this study were considered a measure of the fabric porosity combined with the micro-voids and macro-voids in the fabrics. 

By comparing fabric pore diameters (Figure 2) measured in this study with calculated porosity conducted in previous studies [30,31], similar results were observed, i.e., sheath/core yarn structure with quadrilobal PET and Lyocell fibers in this study exhibited the highest pore diameter, and followed in order by quadrilobal PET/bamboo spun yarn structure, which was in accordance with calculated porosity examined in previous studies [30,31]. These results suggested that the pore diameters according to various yarn structures measured in this study might be applied to examine the moisture absorption and drying properties of high-performance fabrics because the measured pore diameter showed the same trend as the calculated porosity.

Therefore, the wicking and drying rates measured in this study were compared and discussed in terms of the pore diameter and SEM images of the void and capillary channels formed according to the different yarn structures. In addition, the wicking and drying rates of the fabrics with different yarn structures were examined using different evaluation methods for the wicking and drying properties, as shown in the next session.

### 3.2. Absorption Characteristics of the Fabric Specimens

#### 3.2.1. Drop Test by Transverse Wicking Evaluation

The primary concern of this study was to examine how the moisture absorption and drying properties of eco-friendly fiber-embedded fabrics were affected by the yarn structure and fiber characteristics with their evaluation methods. Wicking evaluation in this study was divided into two methods: transverse and vertical wicking. The transverse wicking rate (second) was assessed using the drop test, and the vertical wicking length (mm) was measured using the Bireck method. 

**Figure 3:**
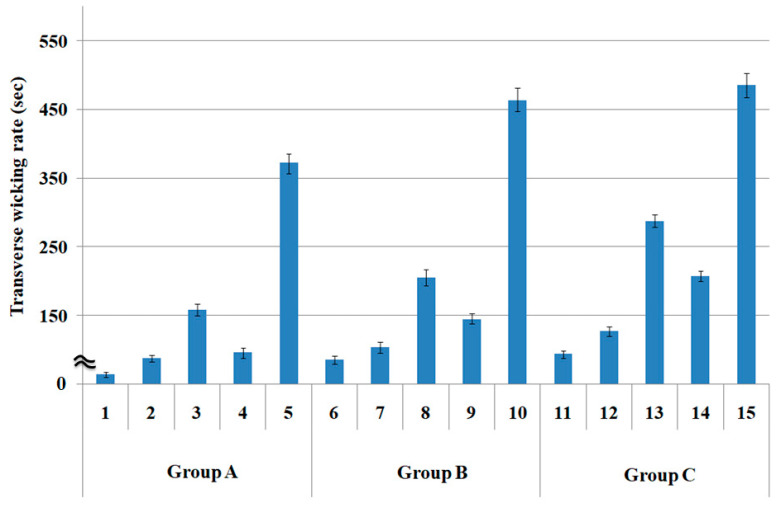
Transverse wicking rates of the fabric specimens (*p* < 0.05).

As explained previously in Table 3, the mean value of the three groups for the transverse wicking rate (second) was statistically significant: as F_0_ (V/Ve) > F(4, 20, 0.95) and *p* < 0.05. Similarly, vertical wicking length (mm), drying rate (min) at a steady state, and drying rate (g) at a transient state were similar to the transverse wicking rate. Figure 3 presents the transverse wicking rates (s) of the specimens measured using the drop test method.

Comparing transverse wicking rate according to warp and weft yarn structures, fabric specimens 1, 6, and 11 with quadrilobal PET/Lyocell sheath/core yarn in the filling direction exhibited superior wicking property (lowest wicking rate), as shown in Figure 3. These results suggest that many voids and capillary channels with a large pore diameter in the sheath/core yarns (yarn specimen (4) in the table [1]) allow the moisture absorbed by the Lyocell wrapper fibers to move out easily via the non-circular cross-sectioned filaments in the core. This result was verified by the largest pore diameter of specimens 1, 6, and 11 (Figure 2). This result was in accordance with the prior study [11], which reported that the wicking by the capillary flow between the filaments increases with increasing fine micro-pore (i.e., pore diameter) developed by a non-circular cross-section of filaments. 

In addition, of fabric specimens 2, 3, and 4 (group A), quadrilobal PET/bamboo spun fabric specimen 2 exhibited a superior wicking rate, followed in order by bamboo spun fabric 4 and PET/Lyocell Siro-fil fabric 3, which was consistent with fabric specimens 7 to 9 (group B) and 12 to 14 (group C). These results were attributed primarily to the larger pore size of the fabric specimens 2, 7, and 12 than that of the other fabric specimens (Figure 2) and partly to the higher moisture retention of the constituent bamboo fibers than that of the Lyocell and PET in the yarns [38], resulting in the better wicking property of the quadrilobal PET/bamboo spun fabric than that of PET/Lyocell Siro-fil fabric. This result was consistent with the previous finding [39]. Das et al. [39] reported that bulky staple yarn fabric with high porosity showed good wicking properties because of the large capillary space due to the non-circular cross-section of constituent fibers incorporated in the bulky yarns.

In addition, PET filament fabric specimen 5, among the five fabric specimens (1 to 5), showed the highest transverse wicking rate, i.e., inferior wicking property, even though it has a large pore size, as listed in Table 3 and Figure 2. This is because the PET filaments are hydrophobic and exhibit low hygroscopicity, i.e., they do not absorb perspiration. Furthermore, the hydrophobic PET was not wetted, which is responsible for the inferior wicking property of the PET filament fabric specimen 5. Fabric specimens 10 (group B) and 15 (group C) showed the highest wicking rate, i.e., inferior wicking property, which can be explained by the same trend as fabric specimen 5. 

In addition, concerning the transverse wicking rate according to the warp yarn structure (Figure 3), the transverse wicking rates of group A were lower than those of groups B and C. Hence, superior wicking property was observed, which was caused by the larger pore diameter because of the sheath/core yarn structure of the warp yarns of group A fabric specimens (1 to 5).

Absorption property (Figure 3), according to yarn structure by the drop test method, might be summarized that the sheath/core yarn and non-circular cross-sectional fiber incorporated bulky yarn fabrics showed superior wicking property to the Siro-fil yarn fabric because of the larger pore diameter and higher water retention of the non-circular cross-sectional fibers in the yarns. These results were consistent with the previous study [30] conducted using the same drop test method as this study, in which similar results to those obtained in this study were appreciated; however, these were explained by the higher calculated porosity of the sheath/core and bulky yarn fabrics than Siro-fil compact yarn fabric, not measured pore diameter applied in this study. 

#### 3.2.2. Bireck Test by Vertical Wicking Evaluation

**Figure 4:**
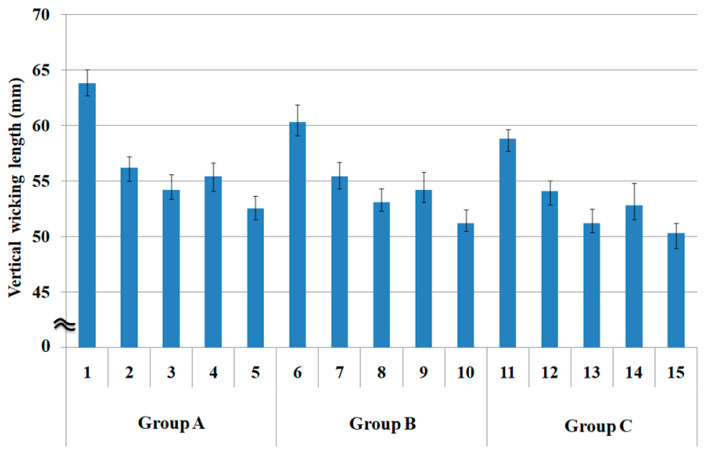
Vertical wicking length of the fabric specimens (*p* < 0.05).

Figure 4 shows the vertical wicking length (mm) of the fabric specimens measured using the Bireck method. As shown in Figure 4, of the five fabric specimens (1 to 5), the vertical wicking length (mm) of the quadrilobal PET/Lyocell sheath/core fabric (specimen 1) showed the highest value, i.e., it was superior to the other fabric specimens. This result was attributed to the greater pore size of the quadrilobal PET/Lyocell sheath/core fabric (specimen 1) (Table 3 and Figure 2) than that of the other specimens; in other words, the voids and capillary channels between the quadrilobal PET filaments in the core and the Lyocell wrapper fibers in a sheath afford an effective capillary radius and allow absorbed moisture to move upward easily.

Of fabric specimens 2, 3, and 4, quadrilobal PET/bamboo spun fabric specimen 2 showed a slightly higher wicking length than specimens 3 and 4. This observation was attributed to the larger pore size (Figure 2) because of the non-circular cross-section of quadrilobal PET and higher hygroscopicity of bamboo fibers in the quadrilobal PET/bamboo yarns of fabric specimen 2 than the other fabric specimens. 

On the other hand, the Siro-fil fabric specimen 3 exhibited a lower wicking length because of the small pore size (Figure 2) and compact yarn structure by the Siro-twist in the yarn specimen (6), which was called spiral wicking in a previous study [11]. Siro-twist can impede water flow in the yarns and fabrics, showing a similar trend to that obtained from the drop test method mentioned previously. This result was in accordance with a prior study [40]. Das and Ishtiaque reported that spiral wicking provides a hindrance to water flow due to the lack of a proper channel or capillary for the water to pass through as well as the lack of pores for holding the water inside the yarns.

PET filament fabric specimen 5 exhibited inferior wicking properties (the lowest wicking length among the five fabric specimens), even though it has a large pore size, as shown in Figure 2. This result was attributed to the hydrophobic characteristics and low hygroscopicity of the PET filaments, which are responsible for the lowest wicking length, as mentioned previously in transverse wicking. The wicking and absorption properties according to the weft yarn structure in the group B and C fabrics showed the same trend as the group A fabric. 

Vertical wicking length (Figure 4) according to yarn structure by Bireck test method was noted that the sheath/core yarn and bulky spun yarn fabrics with a non-circular cross-section of the constituent fibers showed higher values (superior wicking property) than the compact yarn fabric, due to the larger pore diameter and higher hygroscopicity of the constituent fibers in these yarns, which was in accordance with a previous study [31] carried out using the same Bireck test method as in this study. In a previous study, however, the reason for the superior wicking of these yarn structures was explained by the higher calculated porosity, not the measured pore diameter used in this study. In addition, current findings (Figure 4) were consistent with the previous results measured by the same Bireck test methods [22], in which the knitted fabrics with the ring, compact, and air vortex yarn structures were produced using poly trimethylene terephthalate (PTT), wool, and modal fibers. Air vortex yarn structure showed superior wicking properties due to the high porosity of air vortex yarns. Air vortex yarns have many air voids and capillary channels between the parallel fibers in the core and the wrapper fibers in the sheath in the yarns, which is similar to the sheath/core yarn structure in the current study; accordingly, the wicking property due to the sheath/core yarn structure in the current study might be compared with that due to the vortex yarns structure in a previous study [22]. 

In contrast, different findings were reported by another study [21], in which yarn specimens were produced from the cotton/PTT/tencel fibers. Air vortex yarn fabric similar to the sheath/core yarn structure used in this study showed inferior wicking properties, which was in contrast to the current finding. This was attributed to the difference between wool and cotton fiber characteristics, i.e., wool fiber has an elliptic cross-section and bulky shape, but the cross-section of cotton fiber is kidney shape and not bulky. According to previous studies [41,42] related to the absorption of fabrics measured using a moisture management tester (MMT) method, the water absorbed in cotton-incorporated yarn tends to be retained due to the bonding sites for water molecules in the kidney-shaped cross-section of the cotton fibers and fine voids or pores, which prevents the water absorbed from drying out, resulting in a lower spreading speed of the absorbed water and a poor wicking property of the cotton blended yarn fabrics. These results support the importance of the constituent fiber’s bulky characteristics and its cross-sectional shape to the absorption (wicking) of the fabrics.

In addition, regarding the vertical wicking length according to warp yarn structure (Figure 4), the vertical wicking length of the group A fabrics (specimens 1 to 5) was greater than those of groups B (specimens 6 to 10) and C (specimens 11 to 15). That is, the superior wicking property was attributed to the sheath/core yarn structure of the warp yarns of group A fabric specimens. Finally, consistency was noted between the two evaluation methods. Hence, the results obtained through transverse and vertical wicking evaluations of the eco-friendly fiber-embedded fabrics with different yarn structures showed a similar trend.

### 3.3. Drying Characteristics of the Fabric Specimens

#### 3.3.1. Drying Rate (min) at a Steady State

The drying characteristics were also considered using two measuring methods: drying rate (min) at a steady state and drying rate (g) at a transient state after 10 min since the fully absorbed fabric specimens were removed from the water bath.

**Figure 5:**
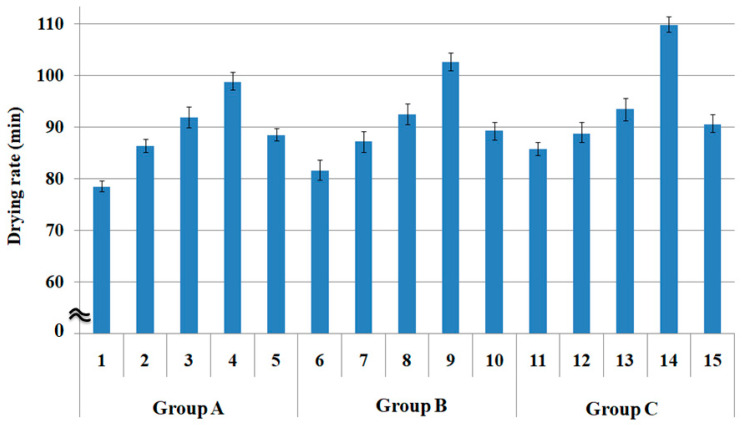
Drying rate at a steady state of the fabric specimens (*p* < 0.05).

Figure 5 presents the drying rate (min) of the fabric groups A, B, and C. The drying rate of the quadrilobal PET/Lyocell fabric 1 in group A was lower than that of the other fabrics (specimens 2 to 5) (i.e., superior drying property). This observation was attributed to the more capillary channels with a larger pore size (Figure 2) in the sheath/core yarns of fabric 1. The capillary channels between the Lyocell fibers in the sheath and quadrilobal PET filaments in the core, as observed by SEM in Table 6 [1] yarn specimen (4), enabled absorbed moisture through the sheath to evaporate from the fabric, which provided more moisture and vapor transmission, and finally resulted in a shorter drying rate in a steady state of fabric 1. 

In contrast, quadrilobal PET/bamboo spun fabric 2 (compared to 3, 4, and 5) showed relatively good drying properties, which was also caused by the large pore size (Figure 2) and many micro-voids (yarn specimen (5) in Table 6 [1]) in the yarn because of the non-circular cross-section of quadrilobal PET fibers, which provide drainage channels to remove and evaporate the perspiration and vapor absorbed from the human body. 

On the other hand, the drying rate of the PET/Lyocell Siro-fil fabric 3 was relatively high, i.e., it exhibited a poor drying property. This observation was attributed to the small pore size because of the Siro-fil twist. The compact yarn structure in PET/Lyocell Siro-fil yarn (6) was shown in the SEM and optical microscopy images (Table 6 [1]), and small pore size was also shown in the PET/Lyocell Siro-fil fabric 3 (Figure 2). These results are consistent with previous findings [43,44]. They showed that the drying property of the fabric became increasingly inferior with decreasing pore size. Bamboo spun fabric 4 exhibited the highest drying rate (i.e., the most inferior drying property), which was attributed mainly to the small pore size in the bamboo spun fabric specimen 4 (Figure 2), and partly to the high moisture regain (30.2% in Table 3) of bamboo spun fabric specimen 4 This observation was attributed to the high absorption characteristics (high water retention) of the bamboo fibers [45], which is in accordance with previous findings [43,44]. They reported that the retained moisture in the compact yarns is prevented from leaking out through the small voids in the yarns, resulting in poor drying properties. In particular, Fangueiro et al. [43] reported that the drying rate of the fabric increased with decreasing the porosity, i.e., small pore size. In addition, the water absorbed from the Siro-fil yarns was prevented from moving upward and horizontally due to the fewer voids and capillary channels in the compact Siro-fil yarns, which resulted in a longer drying rate (i.e., a poor drying property).

In addition, hi-multi PET filament fabric 5 exhibited relatively good drying properties (i.e., low drying rate) compared to Siro-fil fabric 3 and spun fabric 4 (Figure 5), which was caused by the larger pore size of fabric 5 (Figure 2) than that of the Siro-fil and bamboo spun fabrics (specimens 3 and 4), which showed a little different trend compared with the wicking phenomena. A similar trend to the drying rate (mm) according to the fabric group A (1 to 5) was observed in groups B (6 to 10) and C (11 to 15). In addition, the drying rate (min) according to the warp yarn structure showed a similar trend to the wicking property, as mentioned previously.

Overall, by comparing the drying rate at a steady state (Figure 5) obtained in this study with the results in the previous studies [30,31], current results (Figure 5) were consistent with the previous results [30,31], in which drying rate (min) was compared with the calculated porosity according to the yarn structure, not the measured pore diameter in this study. The drying rate of the non-circular cross-sectional fiber incorporated sheath/core yarn fabric showed a superior drying rate due to the higher porosity of the sheath/core yarn fabrics with non-circular cross-sectional fibers. Bamboo yarn fabric spun with non-circular cross-sectional fibers exhibited a relatively superior drying rate to the compact structured yarn fabrics such as Siro-fil and Siro-spun yarns, which were similar to the current results conducted in this study. In addition, according to the other previous studies [21,22] conducted using air-vortex yarns knitted fabric, which was similar to sheath/core yarn fabric used in this study, the drying rate (min) of the air-vortex yarn knitted fabric was shorter and better than those of the ring and compact yarns knitted fabrics. This was attributed to the air-vortex yarn structure with parallel fibers in the core and wrapper fibers in the sheath, which resulted in rapid vapor transmission by diffusion and, finally, in a shorter drying rate of the air-vortex fabrics compared to the ring and compact, knitted fabrics. 

#### 3.3.2. Drying Rate (g) at a Transient State

**Figure 6:**
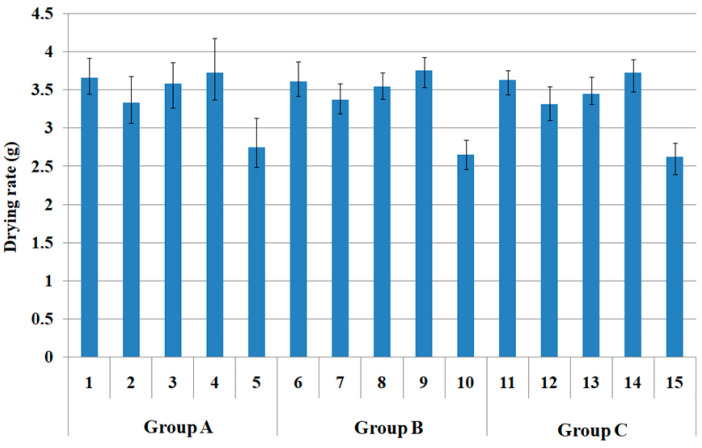
Drying rate at a transient state of each fabric group (*p* < 0.05).

Figure 6 presents the drying rate (g) of each fabric assessed after 10 min since its removal from the water bath.

Hi-multi PET fabric 5 exhibited the lowest drying rate (i.e., superior drying property) and the highest value (i.e., inferior drying property) in bamboo spun fabric 4. In addition, quadrilobal PET/bamboo spun fabric 2 showed a relatively low drying rate (i.e., good drying property). These results were attributed to the larger pore diameter because of yarn structure and partly to the lower moisture regain (M, %) of the fabrics determined by the yarn structure and absorption characteristics of the fibers in the yarns. These were verified by the higher pore diameter (Figure 2) and lower moisture regain (Table 3) of fabric specimens 5 and 2, which gives a lower drying rate (g). In contrast, bamboo spun fabric 4 had a relatively small pore size (Figure 2) and high moisture regain (30.2 % in Table 3), which imparted a high drying rate (i.e., poor drying property), which differed from the results (Figure 5) measured in the steady state. These results suggest that the drying rate (g) determined in the transient state was dependent mainly on the moisture regain and appropriate pore size in the fabrics. 

A similar trend to the drying rate (g) according to the fabric group A (1 to 5) was observed in groups B (6 to 10) and C (11 to 15). In contrast, the drying rate (g) among groups A, B, and C according to warp yarn structure did not show any tendency. These results suggest that the drying rate (g) measured in a transient state differs from the general drying characteristics of eco-friendly fiber-embedded fabrics. Furthermore, the results obtained in this study between drying rate (min) at a steady state and drying rate (g) at a transient state exhibited different trends, and the current result in this study can be compared with previous results [46]. Yanilmaz et al. [46] examined the wicking, wetting, and drying properties of acrylic knitted fabrics using calculated porosity and pore size. Drying capability in their study was assessed through water evaporating rates (WER), which is the same as drying rate (g) at a transient state in this study. They reported that WER decreased with the increase in compactness and the decrease in air space, which means that the drying property is superior in compact fabrics with high porosity and small pore size. This result indicates that compact Siro-fil and bamboo spun fabrics show superior drying properties, which differs from the results of drying rate (min) at a steady state, i.e., this result corresponds to the current finding mentioned above. 

Finally, summarizing the eco-friendly fibers embedded yarn structure required improving the absorption and drying properties in high-performance fabrics, and quadrilobal PET/Lyocell embedded sheath/core yarn fabric and quadrilobal PET/bamboo spun yarn fabric exhibited superior absorption and drying properties. Therefore, Lyocell and bamboo fibers with quadrilobal PET using sheath/core yarn structure are available for the market application of high-performance fabrics, which can contribute to environmental improvement by increasing the content of eco-friendly fibers such as Lyocell and bamboo in the textile industry.

### 3.4. Correlation and Regression Analysis between Wicking and Drying Properties with Pore Diameter and Moisture Regain of the Fabrics

The effects of the yarn structure and constituent fibers’ characteristics on the moisture absorption and drying properties of the fabrics are essential for characterizing the wear comfort characteristics of eco-friendly fiber-embedded fabrics to design high-performance fabrics. Therefore, correlation and regression analysis were conducted to estimate the relationship between absorption and drying properties with pore diameter and moisture regain of fabrics. Table 4 lists the correlation coefficient between each parameter.

As shown in Table 4, a relatively high correlation coefficient (−0.751) between vertical wicking length (W_2_, mm) and transverse wicking rate (W_1_, second) was observed as an inverse correlation. A high correlation coefficient (−0.850) was observed between the drying rate (D_2_, g) and transverse wicking rate (W_1_, second). According to the previous study [34], the correlation coefficient between transverse wicking rate and vertical wicking length was very high −0.98), suggesting that the two tests give equivalent measurements of wickability. In this study, however, it was lower than previous study [34] at −0.751, which was attributed to the difference in the fibers and yarn specimens used, i.e., staple spun yarns with regular fibers (acrylic, PP, PET, and wool) in the previous study [34] and sheath/core and Siro-fil yarns with eco-friendly fibers in this study. 

On the other hand, the relatively high correlation coefficient −0.850) between the drying rate (D_2_, g) and transverse wicking rate (W_1_, second) may be explained by the results reported elsewhere [38]. Lomax [38] reported that drying is associated with the diffusion of moisture absorbed in the yarns and fabrics, which was affected by the absorption and hygroscopicity of constituent fibers. Accordingly, the drying rate (D_2_) may be highly correlated with the wicking rate (W_1_) of the fabrics. 

In the present study, multiple regression analysis was carried out on the effect of pore diameter, the hygroscopicity of the constituent fibers, and fabric drying characteristics on fabric wickability. Table 5 provides the regression equation and determination coefficient (R^2^) of the multiple linear regression analysis. Two multiple regression equations obtained were significant at the 99% confidence level, respectively.

The wickability of the fabrics was strongly dependent on the pore diameter (P), moisture regains (M), and drying property (D_1_ and D_2_) of the fabrics with determination coefficients of 0.92 and 0.86 (Table 5), which means that the wickability of the eco-friendly fiber embedded fabrics is affected by the yarn structure (P) and constituent fiber characteristics (M) and the drying characteristics at a steady state (D_1_) and at a transient state (D_2_). In addition, these results suggest that the wicking property of the fabrics made from various yarn structures using eco-friendly fibers was strongly associated with the drying characteristics, which was verified by a high correlation coefficient (−0.85) between transverse wicking rate (W_1_) and drying rate (D_2_), as shown in Table 4. These results may be compared with a previous study [22]. Fangueiro et al. [22] reported that quadrilobal PET showed good wicking and drying properties, and the wicking length of fabrics is mainly determined by the effective capillary pore distribution and pathway related to yarn structure; in contrast, the drying rate was related to the macromolecular structure of the fiber. These results demonstrate the findings obtained in this study shown in Table 5; that is, wicking length is correlated with the pore diameter related to the yarn structure and drying properties.

## 4. Conclusions

This study examined the moisture absorption and drying characteristics of eco-friendly fiber-embedded high-performance fabrics. Seven types of composite yarns were made from quadrilobal PET, Lyocell, and Bamboo fibers using different types of spinning frames, and PP and PET filaments were prepared to make composite yarns. Fifteen fabric specimens were fabricated using nine composite yarns. The absorption and drying properties of these fabrics were measured using two evaluation methods and discussed in terms of the pore diameter and absorption characteristics of fibers in the yarns. 

The sheath/core yarn structure was critical in improving the absorption and drying properties of eco-friendly fiber-embedded high-performance fabrics. The hygroscopicity and non-circular cross-section of constituent fibers in the yarns affected the absorption and drying properties of the eco-friendly fiber-embedded fabrics. In particular, a similar trend between transverse and vertical wicking evaluation methods in the moisture absorption property was observed. The measured drying property according to the evaluation method between drying rate (min) at a steady state and drying rate (g) at a transient state showed a different trend. Multiple regression analysis showed that the vertical wicking length (mm) and transverse wicking rate (s) of the fabric specimens evaluated in this study were mainly associated with the pore diameter and moisture regain of fabrics (which depends on cross-sectional shape and hygroscopicity of constituent fibers), and were strongly correlated with drying characteristics of the fabrics. 

The superior moisture absorption and drying properties of the eco-friendly high-performance fabrics were observed in the quadrilobal PET/Lyocell sheath/core and quadrilobal PET/bamboo spun fabrics. In contrast, the PET/Lyocell Siro-fil, bamboo spun, and hi-multi PET fabrics exhibited inferior moisture absorption and drying properties. Based on the moisture absorption and fast-drying properties of the eco-friendly high-performance fabrics, the market application of the sheath/core yarn structure using Lyocell and bamboo fibers with quadrilobal PET was available for producing high-performance fabrics. In addition, an increase in eco-friendly fibers with a decrease in synthetic fibers in the textile industry can reduce environmental pollution and improve wear comfort related to moisture absorption and fast-drying properties while wearing clothing made from these fibers.

## Figures and Tables

**Table 1:** Detailed specification of yarn specimens.

Yarn Specimen No.	Yarn Type	Blend Ratio (%)	Spinning m-Method	Yarn No. (d)	Twist		Mechanical Property	
					TM (tpi/ 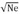 )	Spindle (rpm)	Strength (CN)	Breaking Strain (%)
(1)	PP/Lyocell	PP:39.3 T: 60.7	Sheath/core	132.9	4.53	7000	247.5	8.8
(2)	PET/Lyocell	P: 44.4 T: 55.6	Siro-fil	132.9	4.12	9000	349.3	12.9
(3)	Lyocell	T: 100	Siro-spun	132.9	4.42	11,000	333.2	7.1
(4)	Quadrilobal PET/ Lyocell	Q: 39.3 T: 60.7	Sheath/core	177.2	4.34	9000	253.6	14.8
(5)	Quadrilobal PET/ Bamboo	Q: 48.6 B: 51.4	Ring-spun	177.2	3.82	12,000	376.8	14.1
(6)	PET/Lyocell	P: 44.4 T:55.6	Siro-fil	177.2	4.12	9000	440.5	11.6
(7)	Bamboo	B: 100	Ring-spun	177.2	3.82	12,000	382.6	9.2
(8) *	Hi-multi PET	P: 100	-	75.0	-	-	-	-
(9) *	PP filament	PP: 100	-	100	-	-	-	-

Note: S/F: staple fiber; T: Lyocell; *: existing filament; PP: polypropylene; Q: quadrilobal PET; P: PET; B: bamboo; DTY: draw textured yarn

**Table 2:** Specification of three fabric groups.

Group	Fabric Specimen No.	Warp Yarn	Weft Yarn		Fabric Density (Ends, Picks/cm)		Weight (g/y)	Thickness (mm)
			Yarn 1	Yarn 2	Wp	Wf		
A	1	PP/Lyocell Sheath/core (132.9d)	Quadrilobal PET/ Lyocell S/C (177.2d)	PP (100d)	36.0	24.6	162	0.368
	2		Quadrilobal PET/ Bamboo spun (177.2d)				162	0.345
	3		PET/Lyocell Siro-fil (177.2d)				162	0.341
	4		Bamboo spun (177.2d)				162	0.364
	5		Hi-multi PET (75d)				137	0.352
B	6	PET/Lyocell Siro-fil (132.9d)	Quadrilobal PET/ Lyocell S/C (177.2d)	PP (100d)	36.0	24.6	160	0.396
	7		Quadrilobal PET/ Bamboo spun (177.2d)				161	0.337
	8		PET/Lyocell Siro-fil (177.2d)				161	0.345
	9		Bamboo spun (177.2d)				161	0.345
	10		Hi-multi PET (75d)				137	0.294
C	11	Lyocell Siro-spun (132.9d)	Quadrilobal PET/ Lyocell S/C (177.2d)	PP (100d)	36.0	24.6	158	0.380
	12		Quadrilobal PET/ Bamboo spun (177.2d)				160	0.356
	13		PET/Lyocell Siro-fil (177.2d)				160	0.345
	14		Bamboo spun (177.2d)				161	0.349
	15		Hi-multi PET (75d)				133	0.301

**Table 3:** Pore size, wicking and drying rate of three fabric groups.

Group	Fabric Specimen No.	Pore Diameter P (µm)		Wicking Rate				Drying Rate				Moisture Regain M (%)	
				Transverse Wicking Rate W_1_ (s)		Vertical Wicking Length W_2_ (mm)		D_1_ (min)		D_2_ (g)			
		Mean	Dev.	Mean	Dev.	Mean	Dev.	Mean	Dev.	Mean	Dev.	Mean	Dev.
A	1	3.82	0.100	64.2	1.5	63.8	2.4	78.5	2.1	3.66	0.47	28.3	3.1
	2	3.19	0.088	87.5	2.7	56.2	2.2	86.4	2.6	3.33	0.61	27.2	2.7
	3	2.85	0.112	158.2	4.1	54.2	2.2	91.8	4.1	3.58	0.59	26.4	2.5
	4	2.98	0.110	96.3	1.8	55.4	2.6	98.8	3.5	3.72	0.81	30.2	3.7
	5	3.56	0.124	372.8	9.9	52.5	2.1	88.5	2.5	2.75	0.64	26.3	2.4
B	6	3.20	0.104	86.1	2.5	60.3	2.8	81.6	3.9	3.61	0.45	30.2	3.6
	7	2.85	0.115	103.7	1.8	55.4	2.4	87.2	4.0	3.37	0.39	28.5	3.2
	8	2.30	0.096	205.4	5.2	53.1	2.0	92.4	4.2	3.54	0.35	28.1	2.8
	9	2.70	0.121	144.6	3.7	54.2	2.8	102.6	3.5	3.75	0.39	32.4	3.5
	10	3.11	0.116	463.2	9.3	51.2	2.0	89.4	3.5	2.65	0.39	26.2	2.4
C	11	3.45	0.100	94.3	2.2	58.8	2.0	85.8	2.6	3.63	0.32	34.4	4.2
	12	2.95	0.116	127.6	2.8	54.1	2.2	88.8	3.9	3.31	0.45	34.2	4.5
	13	2.55	0.106	287.2	4.0	51.2	2.1	93.5	4.3	3.45	0.35	34.0	4.0
	14	2.76	0.094	207.1	3.5	52.8	3.3	109.8	3.1	3.72	0.42	36.7	5.1
	15	3.25	0.111	485.6	8.7	50.3	2.3	90.6	3.4	2.62	0.42	32.1	3.8

Note: dev. = max. − min.

**Table 4:** Correlation coefficient between each parameter related to the absorption and drying of the fabrics.

	Pore Diameter (µm)	Transverse Wicking Rate (W_1_)	Vertical Wicking Length (W_2_)	Drying Rate (D_1_)	Drying Rate (D_2_)	Moisture Regain (M)
Pore diameter (P, µm)	1					
Transverse wicking rate (W_1_, s)	−0.482 *	1				
Vertical wicking length (W_2_, mm)	0.547 *	−0.751 *	1			
Drying rate (D_1_, min)	−0.607 *	0.146	−0.566 *	1		
Drying rate (D_2_, g)	−0.281	−0.850 *	0.541 *	0.240	1	
Moisture regain (M, %)	−0.226	−0.112	−0.090	0.474	0.348	1

Note: * significant at the 0.05 level.

**Table 5:** Multiple linear regression analysis of wickability of the fabric specimens.

Wicking (y)	Regression Equation	R^2^	*p*-Value
Vertical wicking length (mm)	y = 38.09 + 4.97 P − 0.16 M −0.185 D_1_ + 6.93 D_2_	0.92 **	1.76 × 10^−5^ < 0.01
Transverse wicking rate (second)	y = 1021.2 + 20.0 P − 5.33 M −5.79 D_1_ + 421.8 D_2_	0.86 **	2.95 × 10^−4^ < 0.01

Note: P: pore dia. (µm); M: moisture regain (%); D_1_: drying rate (min); D_2_: drying rate (g); **: significant at the 0.01 level.

## Data Availability

The data presented in this study are available on request from the corresponding author.
